# The Impact of Amine-Functionalised Iron Oxide Nanoparticles on the Menaquinone-7 Isomer Profile and Production of the Bioactive Isomer

**DOI:** 10.1007/s12033-023-00832-w

**Published:** 2023-07-30

**Authors:** Neha Lal, Mostafa Seifan, Alireza Ebrahiminezhad, Aydin Berenjian

**Affiliations:** 1 School of Engineering, The University of Waikato, Hamilton, 3240 New Zealand; 2 Biotechnology Research Center, Shiraz University of Medical Sciences, Shiraz, Iran; 3 Department of Chemical and Biological Engineering, Colorado State University, Fort Collins, CO 80523 USA

**Keywords:** Menaquinone-7 isomers, Biocompatible iron oxide nanoparticles, 3-Aminopropyltriethoxysilane, L-Lysine, Bacterial cell immobilisation, Fermentation

## Abstract

The K family of vitamins includes a collection of molecules with different pharmacokinetic characteristics. Menaquinone-7 (MK-7) has the finest properties and is the most therapeutically beneficial due to its long plasma half-life and outstanding extrahepatic bioavailability. MK-7 exhibits *cis–trans* isomerism, and merely the all-*trans* form is biologically efficacious. Therefore, the remedial value of MK-7 end products is exclusively governed by the quantity of all-*trans* MK-7. Consumers favour fermentation for the production of MK-7; however, it involves several challenges. The low MK-7 yield and extensive downstream processing requirements increase production costs, resulting in an expensive final product that is not universally available. Bacterial cell immobilisation with iron oxide nanoparticles (IONs) can potentially address the limitations of MK-7 fermentation. Uncoated IONs tend to have low stability and can adversely affect cell viability; thus, amine-functionalised IONs, owing to their increased physicochemical stability and biocompatibility, are a favourable alternative. Nonetheless, employing biocompatible IONs for this purpose is only advantageous if the bioactive MK-7 isomer is obtained in the most significant fraction, exploring which formed the aim of this investigation. Two amine-functionalised IONs, namely 3-aminopropyltriethoxysilane (APTES)-coated IONs (IONs@APTES) and L-Lysine (L-Lys)-coated IONs (L-Lys@IONs), were synthesised and characterised, and their impact on various parameters was evaluated. IONs@APTES were superior, and the optimal concentration (300 $\upmu$g/mL) increased all-*trans* MK-7 production and improved its yield relative to the untreated cells by 2.3- and 3.1-fold, respectively. The outcomes of this study present an opportunity to develop an innovative and effective fermentation method that enhances the production of bioactive MK-7.

## Introduction

The vitamin K series encompasses a range of structurally similar fat-soluble molecules. Vitamin K1 or phylloquinone (PK) and vitamin K2 or menaquinones (MKs) are the prevalent types of dietary vitamin K that have nutritional value for humans [1]. Both PK and MKs contain a 2-methyl-1,4-naphthoquinone constituent, but the structure of the isoprenoid group at the 3-position is unique for each isoform [2]. PK is an individual molecule that is ubiquitous in the chloroplasts of plants and algae that carry out photosynthesis [3, 4]. Hence, it can be readily obtained from numerous dietary sources, including green vegetables (e.g. spinach, broccoli, and iceberg lettuce), plant oils (e.g. olive, cottonseed, canola, and soybean), and products derived from vegetable oils (e.g. salad dressings, margarines, and spreads) [5, 6]. In contrast, MKs consist of a group of molecules with distinct side chains, which can be described by the script MK-*n* (*n* is commonly between four and thirteen and symbolises the number of repeating isoprene units) [7, 8]. MKs are mostly of microbial origin and function as electron acceptors in the electron transport system; thus, they are found in specific dairy, animal, and fermented goods but at low concentrations [6, 8, 9].

All vitamin K subtypes are essential cofactors for the $\upgamma$-glutamyl carboxylase (GGCX) enzyme and play a role in the activation of both hepatic and extrahepatic vitamin K-dependent proteins (VKDPs) [10]. VKDPs participate in several key metabolic pathways, such as the blood coagulation cascade, increasing bone mineralisation, and preventing arterial calcification, which are the primary health gains linked to the sufficient intake of vitamin K [8, 10–12]. Additionally, recent research has revealed that vitamin K consumption also contributes to reducing the risk of various other illnesses and globally relevant diseases, including type 2 diabetes mellitus, Parkinson’s disease, cancer, neurological illnesses, immune disorders, chronic kidney disease, and obesity, and improving the recovery and outcomes of coronavirus disease 2019 (COVID-19) [1, 13–22].

As a result of its long plasma half-life and excellent extrahepatic bioavailability, MK-7 has the greatest efficacy and superior therapeutic value among all K vitamers [23, 24]. However, the dietary sources of MK-7 are limited, and it occurs in small amounts in foods that interest mainstream consumers [25, 26]. This has heightened the demand for MK-7 dietary supplements and nutraceuticals with widespread appeal to accompany natural foods and satisfy the daily intake needs of all populations.

**Fig. 1:**
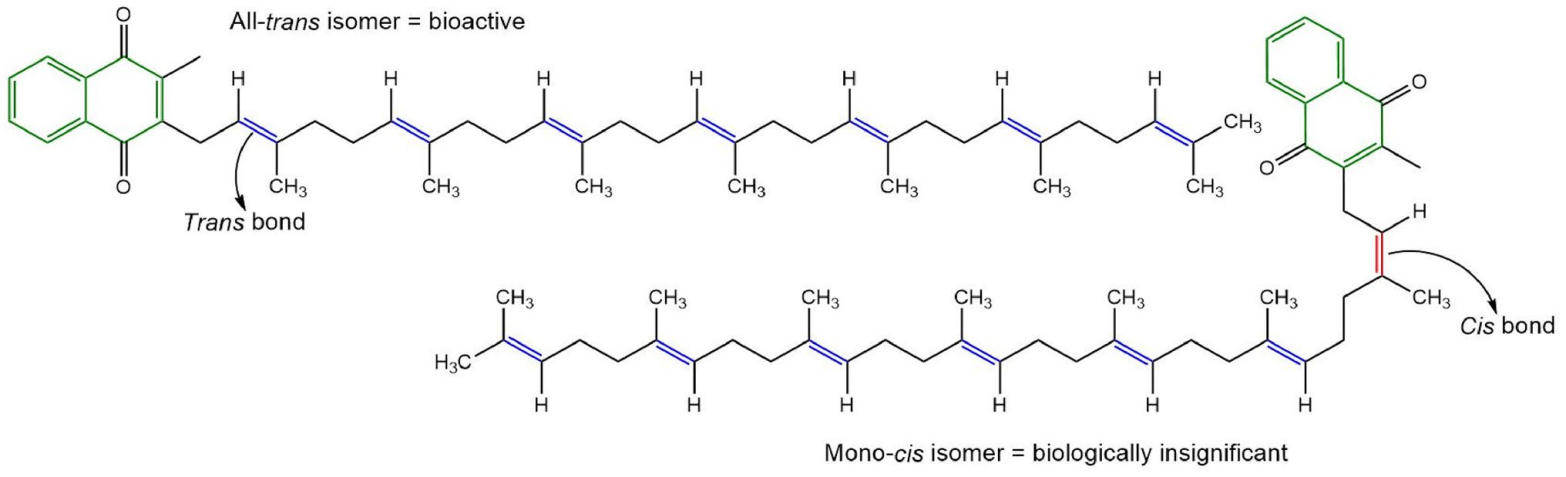
The double bond arrangement and resulting molecular structure of the all-*trans* and *cis* geometric isomers of MK-7

It must be appreciated that, like many natural molecules, MK-7 exhibits geometric isomerism. The all-*trans* isomer is biologically significant, and the *cis* isomers have negligible bioactivity [8, 27, 28]. The all-*trans* isomer has a linear organisation (Fig. 1) due to the *trans* bond arrangement. In contrast, the *cis* isomers, which contain one or more *cis* double bonds, have a non-linear shape (Fig. 1). The biological activity of MK-7 molecules is dictated by the double bond configuration in the isoprenoid side chain, as it impacts their shape and ability to associate with subcellular structures and perform their biological function [29, 30]. It has been established that the *cis* isomer has reduced carboxylative capacity and biological significance in comparison to all-*trans* MK-7 [31]. Therefore, consideration of the amount of the different geometric isomers in MK-7 consumer products is vital, as only the all-*trans* isomer is biologically effective.

Natural fermentation or chemical reaction methods can be used to synthesise MK-7, and the manufacturing process and techniques employed to purify the crude reaction mixture influence the isomer profile of the MK-7 product [28, 32–36]. Although chemical approaches tend to be cheaper, natural synthesis has greater acceptance among consumers. Moreover, fermentation-based production using microorganisms for the industrial synthesis of MK-7 ensures environmental sustainability and, hence, can fulfil the desire of consumers while supporting sustainable development initiatives [37]. Nevertheless, MK-7 production by fermentation is accompanied by many challenges, the major concerns being the low yield of the vitamin and the need for several complex steps and unit operations to satisfy its extensive downstream processing requirements [38, 39]. These drawbacks increase the production cost and result in an expensive final product that is not widely accessible to consumers.

Many studies have aimed to boost the fermentation yield and MK-7 concentration by exploring and optimising various facets of the fermentation process, such as the media composition, fermentation strategy (using liquid-state fermentation (LSF), solid-state fermentation (SSF), or biofilm reactors), operating conditions, and cell treatment methods [40–48]. The outcomes of prior investigations and their associated developments have contributed significantly to the comprehension of the different features of MK-7 fermentation and enhanced the production of this essential K vitamin. However, further opportunities for improvement still exist, and all except our prior studies [41, 47] have not considered the occurrence of MK-7 isomers, accounting for which is paramount in view of their differing bioactivity. Additionally, optimising aspects inherent to the fermentation process alone only has the capacity to increase the production of MK-7 and provides minimal scope to refine the overall fermentation scheme through process intensification. Consequently, there is a demand for new methods to enhance the MK-7 yield achieved from fermentation and/or streamline the production system by decreasing the number of complex unit operations required, in addition to ensuring that the bioactive MK-7 isomer is obtained entirely or in the largest proportion. In this regard, nanomaterials (NMs) show great promise in addressing the limitations of MK-7 fermentation.

NMs contain structural components between 1 and 100 nm in size in at least one aspect [49]. The nanoscale structure increases the surface area-to-volume ratio and provides NMs with unique biological and physicochemical characteristics, which are absent in the equivalent macroscopic material, making them ideal for many novel applications [49–52]. Among the various types of NMs, nanoparticles (NPs) are pertinent to MK-7 fermentation and have the ability to overcome the primary challenges linked to the fermentation-based production of the vitamin. IONs are particularly relevant to MK-7 fermentation and have been employed to immobilise bacterial cells to increase the MK-7 yield and enhance the process output by improving the metabolic productivity of the cells [38, 39, 53]. Furthermore, IONs display superparamagnetism, which can be manipulated to allow cell separation and dispersion with an external magnetic field [38]. This will decrease the number of downstream purification steps and create an opportunity for process intensification. Thus, it is apparent that the use of IONs for bacterial cell immobilisation is an innovative approach to address the obstacles in large-scale MK-7 production.

Previous investigations [38, 39, 53] have explored the ability of bacterial cell immobilisation with IONs to increase MK-7 synthesis and enable process intensification using magnetic separation technology to facilitate cell recovery. In these studies, magnetic IONs were employed to immobilise *Bacillus subtilis natto* cells, and the surface of the cells was decorated through several non-specific bonds. The effect of cell immobilisation on bacterial growth and MK-7 production was also assessed, along with the prospect for in-place product removal and cell reutilisation. It was established that immobilisation with IONs improves both the concentration and yield of MK-7 relative to fermentation with free microbial cells. Moreover, the superparamagnetic nature of IONs allows the separation of bacterial cells using an externally applied magnetic field with a high capture efficiency that is not significantly compromised over successive cycles.

In light of the differing efficacy of MK-7 isomers, using IONs to increase the productivity of MK-7 fermentation and aid process intensification is only advantageous if the all-*trans* isomer is synthesised almost exclusively or in the largest quantity. It is important to understand that all earlier studies have examined MK-7 production without considering the isomer composition obtained from fermentation. Our previous investigation was the first to assess the impact of uncoated IONs on microbial growth and the MK-7 isomer concentration and yield achieved from fermentation [54]. Immobilisation of *B. subtilis natto* cells improved the efficiency of the fermentation process, and a 1.6-fold greater yield of the bioactive isomer was attained at the optimal NP concentration compared to the free cells. However, it has been observed that although naked IONs positively affect MK-7 production, they exhibit low physicochemical stability and toxic effects on microbial cells, inhibiting bacterial growth. This is disadvantageous from the perspective of cell recycling using magnetic separation, as it is essential to preserve the viability and metabolic efficiency of bacterial cells to ensure the effectiveness of this technique.

Therefore, biocompatible coatings like amino acids could eliminate such unfavourable properties. Amino acids are an ideal coating material due to their biocompatibility, chemical stability, and surface activity. Many amino acids are also suitable for human consumption and have been used as biocompatible coatings or constituents in dietary supplements. Thus, amino acid coatings can mitigate the toxic effects of IONs in food-related applications. L-Lys is especially desirable, as it does not have a detrimental impact on the fundamental properties of IONs [39]. It also adds amine functional groups to the structure of NPs, which improves their interaction with negatively charged regions of the bacterial cell membrane and, consequently, increases the potential for surface associations [39]. Another suitable amine-functionalised coating is APTES, which confers the same properties as L-Lys but prevents oxidation of NPs and preserves their crystalline structure [55]. Furthermore, IONs@APTES significantly decrease biofilm formation without impairing cell growth and viability [53]. This is beneficial for industrial MK-7 production, as the formation of biofilms is one of the dominant issues in large-scale fermentation since it leads to many process and operational problems that decrease the performance of the system, reduce the yield and quality of the target product, and increase the process and equipment-related costs. Overall, previous studies suggest that IONs, particularly IONs with biocompatible coatings, are a valuable tool to enhance the production and yield of the vitamin and overcome the challenges accompanying large-scale MK-7 fermentation.

Hence, this study focused on assessing the effect of amine-functionalised IONs on the MK-7 isomer composition obtained from fermentation. Two types of biocompatible NPs, specifically IONs@APTES and L-Lys@IONs, were prepared and analysed using various methods, and the impact of each on microbial growth and MK-7 isomers were evaluated. The conclusions drawn from this investigation will expand the existing knowledge of the ability of biocompatible amino acid-coated IONs to boost the productivity of MK-7 fermentation. This, in addition to the potential for process intensification with the assistance of magnetic separation technology, will likely enable the evolution of a streamlined industrial fermentation process that selectively targets the production of the all-*trans* isomer. A more efficient fermentation system will possibly entail lower production costs and improve the affordability and accessibility of biologically efficacious fermented MK-7 nutraceuticals, functional products, and dietary supplements.

## Materials and Methods

### Chemicals and Materials

The all-*trans* MK-7 (98.1%) analytical standard was obtained from ChromaDex (Los Angeles, CA, USA). FeSO_4_⋅7H_2_O and glucose were supplied by Ajax Finechem Pty Ltd (Taren Point, NSW, Australia), and tryptone and yeast extract were acquired from Becton, Dickinson and Company (Franklin Lakes, NJ, USA). Methanol, ethanol, *n*-hexane, 2-propanol, NH_4_OH, and soy peptone were procured from Merck Millipore (Burlington, MA, USA). CaCl_2_, FeCl_3_⋅6H_2_O, glutaraldehyde, sodium cacodylate, APTES, and L-Lys were purchased from Sigma-Aldrich Co. (St. Louis, MO, USA). NaCl was obtained from a domestic source, and nutrient (BHI) agar plates were acquired from Fort Richard Laboratories (Auckland, New Zealand).

### Microorganism and Inoculum Preparation

*B. subtilis natto* is often utilised in MK-7 fermentation experiments, as well as those involving NPs [38, 39, 53, 54]. Additionally, *B. subtilis natto* enables a high fermentation yield and is classed as generally recognised as safe (GRAS); thus, it is considered suitable for large-scale MK-7 production and is preferably employed for manufacturing MK-7 products [42, 56]. Therefore, it was deemed the most appropriate microbial strain for this investigation. The microbial spore solution was produced using the approach implemented by Berenjian et al. [40]. The *B. subtilis natto* cells were grown in a liquid mixture of NaCl, yeast extract, and tryptone and plated on BHI agar plates. The plates were stored in an incubator at 37 °C for 48 h. The bacterial growth was scraped off the agar plates and submersed in a sterile saline solution. The microbial suspension was then placed in an 80 °C water bath for 30 min to inactivate the growing cells and induce the generation of spores. Centrifugation (laboratory centrifuge, Sigma Laborzentrifugen GmbH, Osterode am Harz, Germany) at 3000 rpm for 10 min was used to isolate the cell debris. The consequent microbial spore suspension acted as the inoculum for the NP fermentation procedures.

### NP Synthesis and Characterisation

#### Synthesis of Uncoated IONs

Uncoated IONs (Fe_3_O_4_) were produced in an inert atmosphere using the co-precipitation technique outlined by Ebrahiminezhad et al. [38]. FeCl_3_⋅6H_2_O (1.17 g) and FeSO_4_⋅7H_2_O (0.74 g) were combined in 50 mL of distilled water, and the solution was briskly agitated for 1 h at 70 °C in a nitrogen atmosphere to avoid oxidation. NH_4_OH (5 mL) was rapidly added to the solution, which was then stirred for a further 1 h until precipitation occurred. After the reaction, the magnetic particles were separated with a permanent magnet. The IONs were washed several times with boiled distilled water to eliminate unwanted compounds before drying for 24 h at 50 °C in an oven (Contherm Thermotec 2000, Contherm Scientific Ltd, Wellington, New Zealand).

#### Synthesis of IONs@APTES

APTES coating was carried out using the methodology proposed by Ebrahiminezhad et al. [55]. Uncoated IONs (0.7 g) were added to a 1:1 (*v/v*) solution of ethanol and distilled water (25 mL) and sonicated (Qsonica-Q800R, Newtown, CT, USA) while kept in an ice bath for 2 min to achieve a homogenous mixture. Afterwards, 2.8 mL of APTES solution was injected into the mixture under nitrogen and vigorously stirred for 2 h at 40 °C. The coated IONs were then collected with a permanent magnet. A mixture of ethanol and distilled water was used to wash the magnetic particles to exclude contaminants before drying for 24 h at 50 °C in an oven (Contherm Thermotec 2000, Contherm Scientific Ltd, Wellington, New Zealand).

#### Synthesis of L-Lys@IONs

The procedure employed by Ebrahiminezhad et al. [39] was used to synthesise the L-Lys@IONs. Accordingly, FeSO_4_⋅7H_2_O (0.74 g), FeCl_3_⋅6H_2_O (1.17 g), and L-Lys (1.6 g) were combined in 50 mL of distilled water, and the solution was robustly mixed at 70 ^∘^C under nitrogen for 1 h. NH_4_OH (5 mL) was successively added to the reaction mixture, which was agitated for another 1.5 h until precipitation occurred. The particles were then magnetically separated, washed with boiled distilled water to extract pollutants, and dried for 24 h at 50 °C in an oven (Contherm Thermotec 2000, Contherm Scientific Ltd, Wellington, New Zealand).

#### Characterisation

The size and morphology of the synthesised NPs were determined by transmission electron microscopy (TEM; Philips, CM 10, Philips Electron Optics, Eindhoven, The Netherlands). A NP dispersion was prepared in distilled water for the TEM analysis, and a drop of the solution was put on a copper grid coated with carbon. All TEM images were taken at HT 100 kV. The identity of important chemical bonds and functional groups was established using Fourier-transformed infrared (FTIR) spectroscopy (Bruker VERTEX 70 FTIR spectrometer, Bruker, Kassel, Germany) in the span of 4000–400 cm^−1^. Before the FTIR procedure, a pellet with a NP-to-KBr ratio of 1% was made and subjected to a hydraulic press for 10 min to create a solid disc. All samples were assessed at room temperature by the FTIR instrument. X-ray powder diffraction (XRD; Siemens D5000, Munich, Germany) was used to evaluate the crystalline structure of the coated NPs. The dried powder was packed on a zero-background silicon holder, and the excess was removed using a brush and straight edge. The analysis was conducted at ambient temperature, 40 mA, and 45 kV, with a step size of 0.0530° and an exploration range (2$\uptheta$) within 20° and 90°.

#### Cell Fixation and Scanning Electron Microscopy (SEM) Analysis

SEM (Hitachi Regulus SU8230 FE-SEM, Tokyo, Japan) was used to visualise the surface structure of the coated NPs and their interaction with the bacterial cells. The bacterial cell samples were prepared following a similar technique to Ebrahiminezhad et al. [38]. A 10 $\upmu$L drop of the sample was put on a coverslip before passing it through the flame of a Bunsen burner to heat-fix the bacterial smear. The sample was then chemically fixed for 45 min by placing the coverslip in 2.5% (*v/v*) glutaraldehyde in 0.1 M sodium cacodylate buffer before rinsing with saline for 15 min. The coverslip was then kept in a series of ethanol concentrations (30, 50, 70, 80, 90, and 95%) for 10 min each to dehydrate the bacterial cells. Afterwards, the sample was stored for 20 min in pure ethanol and dried using a critical point dryer (Polaron E3000, Quorum Technologies, East Sussex, England, UK). The dried microbial samples and pure ION powders were coated with platinum after mounting them on an aluminium stub. SEM images of IONs, free-floating cells, and bacterial cells immobilised with coated IONs were captured at 3 kV.

### Bacterial Cell Immobilisation and Fermentation

The fermentation media, comprising 1% (*w/v*) glucose, 2% (*w/v*) yeast extract, 2% (*w/v*) soy peptone, 2% (*w/v*) tryptone, and 0.1% (*w/v*) CaCl_2_ [41], was made and autoclaved (TOMY SX-700E, Tokyo, Japan) at 121 ^∘^C for 20 min. Following sterilisation, the samples were inoculated with 2% (*v*/*v*) of the bacterial spore suspension. Stock solutions of IONs@APTES and L-Lys@IONs (0.01 g/mL) were individually prepared with sterilised distilled water and added to the samples at various concentrations (0–600 $\upmu$g/mL). The samples for each type of coated NP were prepared separately, and three replicate samples for each concentration were considered. All samples were fermented aerobically at 200 rpm and 40^∘^C for 7 days to enable the coated NPs to attach to the surface of the microbial cells. The fermentation parameters were selected from our earlier investigation [47].

### MK-7 Extraction

Preceding analysis, MK-7 was removed from the fermented samples, as summarised by Berenjian et al. [40]. A solution of *n*-hexane and 2-propanol was added to the samples in a ratio of 1:2 (*v*/*v*) while maintaining a liquid-to-organic ratio of 1:4 (*v*/*v*) before thoroughly mixing for 2 min with a vortex mixer. The different layers of liquid were split by centrifuging (laboratory centrifuge, Sigma Laborzentrifugen GmbH, Osterode am Harz, Germany) the mixture for 10 min at 3000 rpm. The top layer was separated from the remaining liquid and evaporated to isolate the extracted MK-7.

### MK-7 Analysis

MK-7 analysis was performed employing the methodology discussed in our previous report [41]. The MK-7 isomer concentration was assessed with a Dionex high-performance liquid chromatography (HPLC) system (Thermo Fisher Scientific, Waltham, MA, USA), which consisted of a photodiode array UV detector, an automated sample injector, four pumps, and a thermostatted column compartment. Separation was conducted at 40 °C using a COSMOSIL Cholester packed (reversed-phase) column (100 mm × 2 mm × 2.5 $\upmu$m; Nacalai Tesque Inc., Kyoto, Japan). The compounds were eluted isocratically with methanol (mobile phase) at a flow rate of 0.2 mL/min. The injection volume was 10 $\upmu$L, the autosampler temperature was 10 °C, the analytical wavelength was 248 nm, and the run-time was 30 min. Data were collected with the Chromeleon 7 program (Thermo Fisher Scientific, Waltham, MA, USA), and the *cis* MK-7 isomer was ascertained by a relative retention time (RRT) of 1.12. The MK-7 concentration of the samples was deduced from a linear MK-7 standard curve (*R*^2^ = 0.99), which was developed by measuring the area of peaks corresponding to known concentrations of the reference standard.

Liquid chromatography–mass spectrometry (LC–MS) was utilised to validate the identity of the MK-7 isomers and confirm their retention times, as outlined in our earlier study [41]. The LC–MS setup contained a Dionex Ultimate 3000 ultra-high-performance liquid chromatography (UHPLC) apparatus and a QExactive mass spectrometer with a HESI II source (Thermo Fisher Scientific, Waltham, MA, USA). The equipment was controlled with the Thermo XCalibur 4.3 platform (Thermo Fisher Scientific, Waltham, MA, USA), and data handling was accomplished using the Chromeleon 7.3 package (Thermo Fisher Scientific, Waltham, MA, USA). The previously described chromatography conditions were employed to separate the MK-7 isomers using liquid chromatography; however, an injection volume and a run-time of 5 μL and 37 min, respectively, were used to suit the needs of the LC–MS equipment. Data were acquired in the positive ionisation mode with a maximum injection time of 200 ms, a resolution of 70,000, an AGC target of 3 × 10^6^, and a MS1 scan range between 150 and 1000 *m/z*. The mass spectrometry (MS) data were evaluated with the Thermo FreeStyle 1.6 application (Thermo Fisher Scientific, Waltham, MA, USA).

### Cell Density and pH Measurements

Bacterial growth was estimated from measurements of the cell density. The optical density (OD) of the samples was measured using a UV–vis spectrophotometer (Shimadzu UV-1900, Kyoto, Japan) at an analytical wavelength of 600 nm after suitable dilution with distilled water. The pH of the samples was assessed using a laboratory pH meter (CyberScan pH 100, Eutech Instruments, Paisley, UK).

### Statistical Methods

Analysis of variance (ANOVA) was used to determine the statistical significance, and the mean values of different groups were compared with a two-sample* t*-test. All data were described as the mean $\pm$ standard error (SE) of triplicate samples, and statistical significance was accepted at *p* < 0.05.

## Results and Discussion

### Synthesis and Characterisation of Biocompatible Amine-Functionalised IONs

**Fig. 2:**
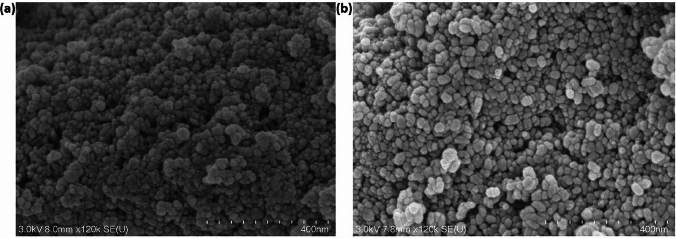
SEM image of the exterior surface structure of the **a** IONs@APTES and **b** L-Lys@IONs

**Fig. 3:**
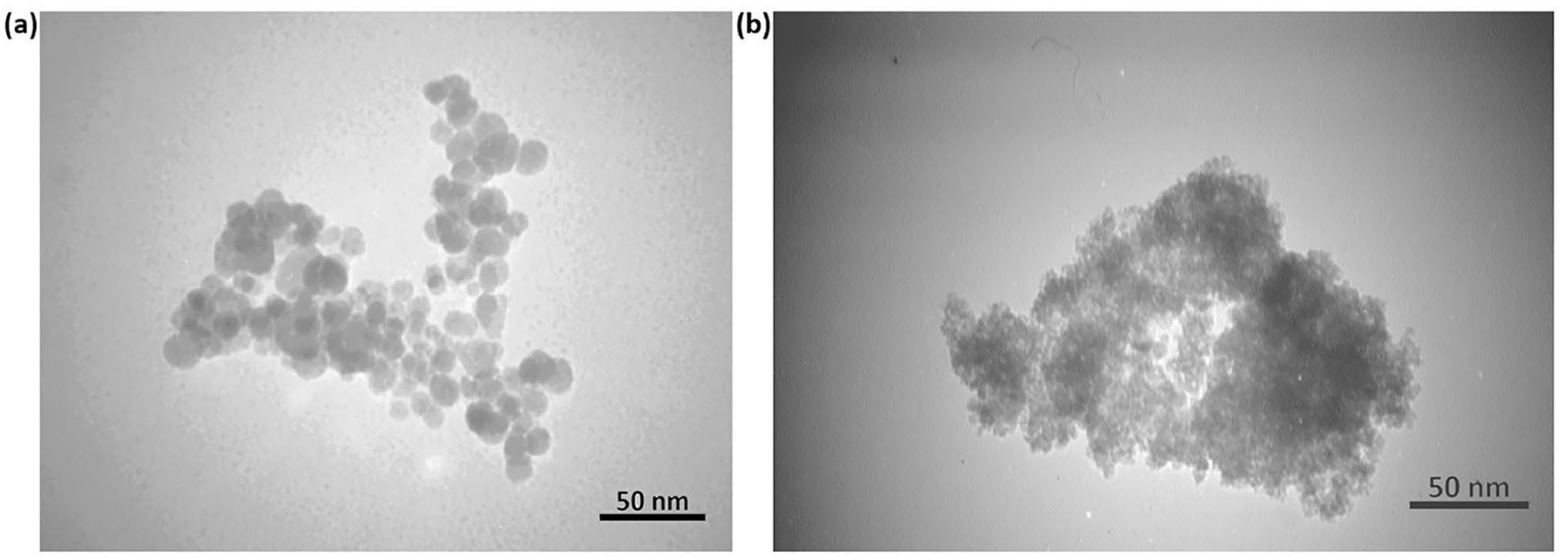
TEM micrograph of the **a** IONs@APTES and **b** L-Lys@IONs

The co-precipitation method was used to synthesise biocompatible amino acid-coated IONs (IONs@APTES and L-Lys@IONs). The SEM images (Fig. 2) and TEM micrographs (Fig. 3) show that both types of amine-functionalised IONs have a spherical morphology. The NPs also have a reasonably uniform size distribution ranging from 7–20 nm for the IONs@APTES and 4–10 nm for the L-Lys@IONs, with an average particle size of 11 nm and 7 nm, respectively.

**Fig. 4:**
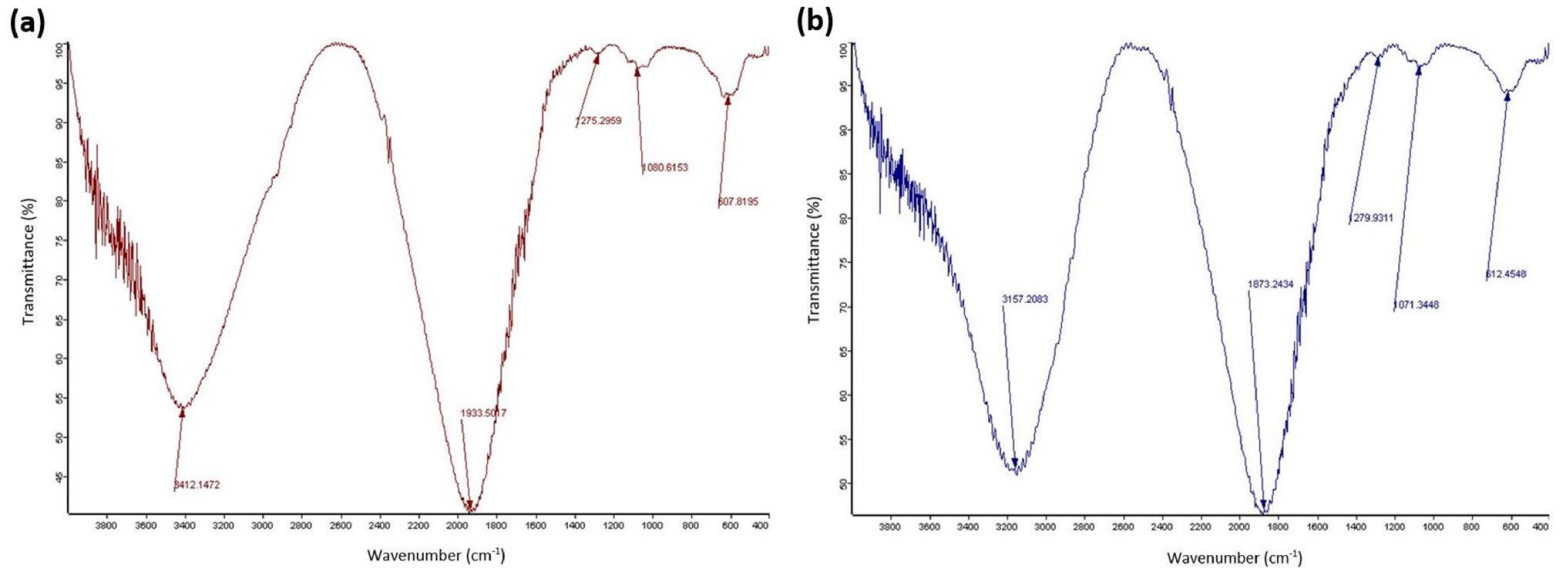
FTIR spectrum of the **a** IONs@APTES and **b** L-Lys@IONs

The FTIR spectra of the coated IONs are provided in Fig. 4 and illustrate the distinguishing Fe–O peaks at around 608 cm^−1^ and 420 cm^−1^ for the IONs@APTES and 612 cm^−1^ and 425 cm^−1^ for the L-Lys@IONs. The peak at approximately 1081 cm^−1^ signifies the stretching vibration of the Si–O bond in the IONs@APTES [53]. The successful coating of IONs with APTES is also indicated by the peaks at about 2850 cm^−1^ and 2900 cm^−1^, which depict the presence of aliphatic -CH_2_ groups and can be ascribed to the symmetric and asymmetric –C–H stretching vibrations, respectively [53]. For the L-Lys@IONs, C–O and C=O stretching vibrations can be visualised at approximately 1440 cm^−1^ and 1630 cm^−1^, respectively, and the peak at roughly 2925 cm^−1^ denotes the overlap between the C-H and N–H stretching vibrations with the O–H stretching vibration [57]. In addition, during the production of IONs using the co-precipitation technique, the unsaturated iron atoms at the surface interact with water molecules and OH^−^ ions in the aqueous medium and modify the surface of the NPs [38, 57]. This is represented by the peaks at about 3412 cm^−1^ and 1934 cm^−1^ for the IONs@APTES and 3157 cm^−1^ and 1873 cm^−1^ for the L-Lys@IONs, which correspond to the O–H bending and stretching vibrations, respectively.

**Fig. 5:**
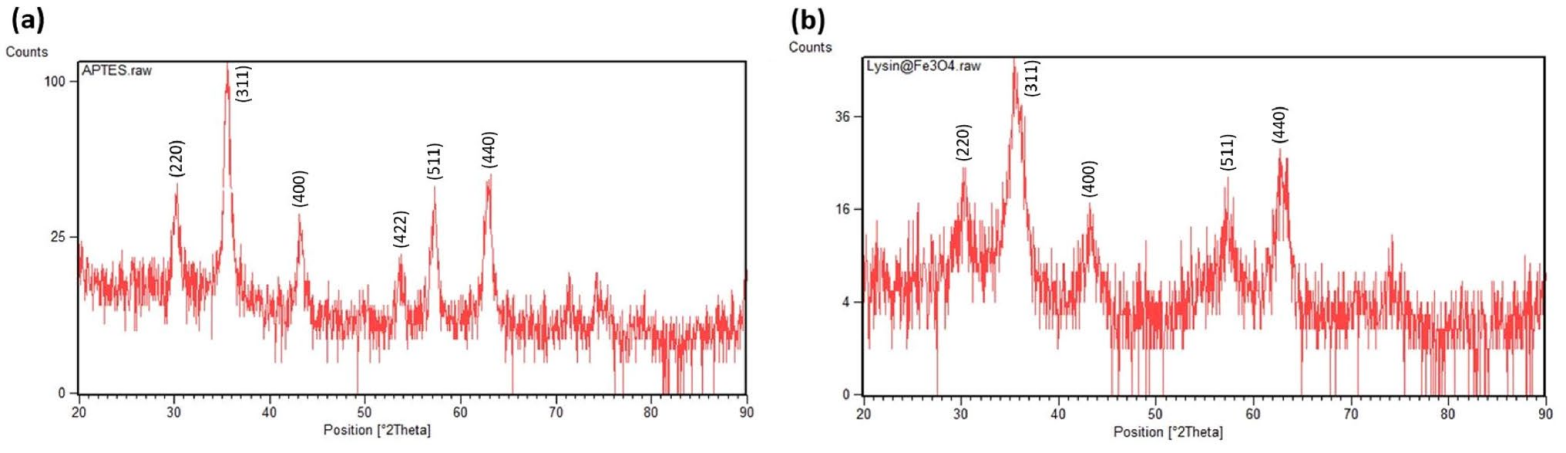
XRD pattern of the **a** IONs@APTES and **b** L-Lys@IONs

The XRD patterns for the amine-functionalised IONs are presented in Fig. 5, and the intensity peaks obtained from the analysis are comparable for the two kinds of coated IONs. The XRD pattern for the IONs@APTES shows definite intensity peaks at 2$\uptheta$ degrees of 30°, 35.5°, 43°, 53°, 57°, and 63°, which represent (220), (311), (400), (422), (511), and (440) Bragg reflections, respectively. Similarly, the XRD pattern for the L-Lys@IONs displays characteristic intensity peaks at 2$\uptheta$ degrees of 30°, 35.5°, 43°, 57°, and 63°, which denote (220), (311), (400), (511), and (440) Bragg reflections, respectively. These distinctive peaks correspond to the crystal structure of magnetite and confirm the production of IONs [39, 53]. The sharper peaks for the IONs@APTES suggest that they have greater crystallinity than the L-Lys@IONs synthesised in this study. However, since the XRD analysis resulted in equivalent peaks for both types of coated IONs, it indicates that amine-functionalisation with biocompatible amino acid coatings does not significantly impact the crystal structure.

### Interaction of Coated IONs with the Bacterial Cell Surface

**Fig. 6:**
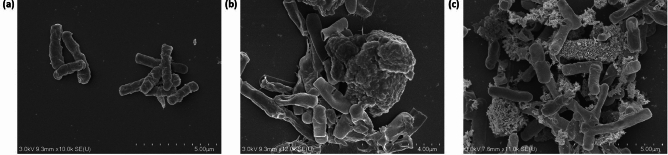
SEM image of the **a** untreated bacterial cells, **b** bacterial cells immobilised with IONs@APTES, and **c** bacterial cells immobilised with L-Lys@IONs

Interactions between the biocompatible IONs and microbial cells were viewed using SEM. The effective decoration and immobilisation of *B. subtilis natto* cells with IONs@APTES and L-Lys@IONs in comparison to the free bacterial cells are depicted in Fig. 6. Both types of amine-functionalised IONs are many orders of magnitude smaller than the microbial cells. The small particle size and large surface-area-to-volume ratio of the coated IONs and the presence of positively charged amine groups allow them to attach to the surface of the bacterial cells through several bonding interactions, including hydrogen bonds, hydrophobic interactions, electrostatic attractions, and Van der Waals forces [39, 53]. As the interactions between the NPs and microbial cells are non-specific, the IONs arbitrarily attach to their surface. Since the interaction between all cells is not consistent, some bacteria are more extensively decorated than others.

Decoration of microbial cells with biocompatible magnetic IONs is advantageous, as it can enable cell removal with an external magnetic field, which creates an opportunity for process intensification by reducing the number of complex downstream processing steps required for the isolation and purification of MK-7. This will streamline the production process and permit the separated bacterial cells to be re-employed in subsequent fermentation batches, decreasing production costs. Nonetheless, to allow the cells to be recycled, it is vital to preserve their growth and metabolic characteristics and ensure that immobilisation with amine-functionalised magnetic IONs has no undesirable effects on cell viability.

### The Influence of Bacterial Cell Immobilisation with Amine-Functionalised IONs on Microbial Growth

**Fig. 7:**
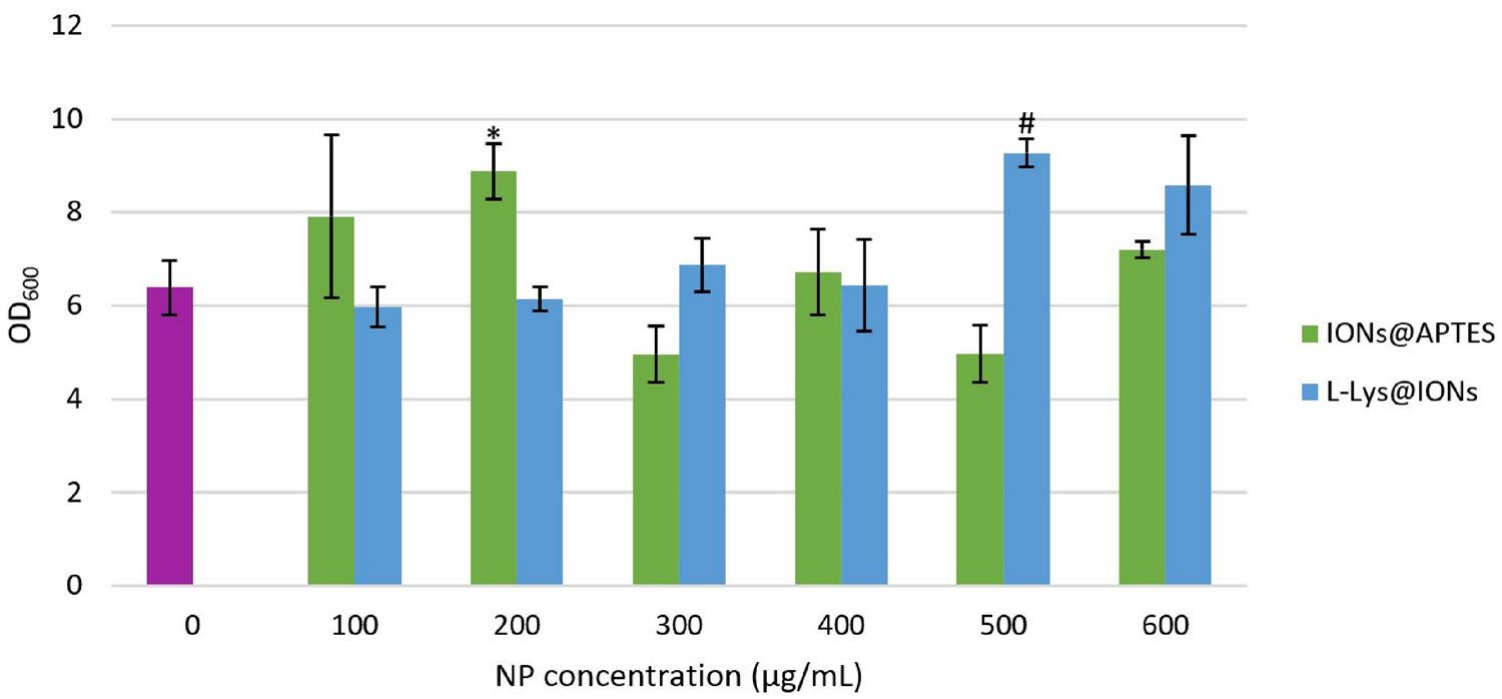
The impact of bacterial cell immobilisation with coated IONs on microbial growth, where * and # indicate a significantly different OD compared to the control (*p* < 0.05) for the IONs@APTES and L-Lys@IONs, respectively

The effect of microbial cell immobilisation with IONs@APTES and L-Lys@IONs on bacterial growth was considered, and it can be seen that the impact on cell growth differs between the two types of amino acid coatings (Fig. 7).

For the IONs@APTES, the OD for the control (6.39), 400 $\mathrm{\upmu g}/$mL (6.72), and 600 $\mathrm{\mu g}/$mL (7.21) groups is comparable. The OD attained for an IONs@APTES concentration of 100 $\mathrm{\upmu g}/$mL (7.91) and 200 $\mathrm{\upmu g}/$mL (8.88) is relatively similar and noticeably higher than the other groups, whereas that for the 300 $\mathrm{\upmu g}/$mL (4.96) and 500 $\mathrm{\upmu g}/$mL (4.97) groups is alike and considerably lower than all other concentration groups. In contrast, for the L-Lys@IONs, the OD obtained for the majority of the investigated concentrations (control, 100 $\upmu$g/mL, 200 $\upmu$g/mL, 300 $\upmu$g/mL, and 400 $\mathrm{\upmu g}/$mL) is fairly consistent (between 5.97 and 6.87), while that for the 500 $\upmu$g/mL and 600 $\mathrm{\upmu g}/$mL groups is appreciably greater than the other L-Lys@IONs concentration groups. Interestingly, the OD achieved at an IONs@APTES concentration of 100 $\upmu$g/mL and 200 $\upmu$g/mL and a L-Lys@IONs concentration of 500 $\upmu$g/mL and 600 $\upmu$g/mL is almost equivalent.

The ANOVA assessment demonstrated that the difference in the OD between all ION concentration groups is not statistically significant for the IONs@APTES (*p* = 0.106) but is statistically significant for the L-Lys@IONs (*p* = 0.038). The OD achieved at each NP concentration for both forms of amino acid coatings was also compared with the control using a *t*-test. It was determined that the OD obtained for an IONs@APTES and L-Lys@IONs concentration of 200 $\upmu$g/mL and 500 $\mathrm{\upmu g}/$mL, respectively, were appreciably higher than the untreated cells (*p* = 0.018 for the IONs@APTES and *p* = 0.004 for the L-Lys@IONs). The ANOVA results also established a statistically significant difference (*p* = 0.028) in the OD measurements between the different concentrations of IONs@APTES and L-Lys@IONs when considered together, implying that the nature of the amino acid coating influences bacterial growth.

The response of microbial cells to immobilisation with magnetic IONs is often variable and tends to differ among Gram-positive and Gram-negative bacterial strains, possibly as a result of the dissimilarities in their cell wall structure, metabolic characteristics, and cellular composition [58]. The exact nature of the interaction between IONs and bacterial cells is determined by several factors, such as the characteristics of the IONs, bacterial species, and culture conditions [59, 60]. Possible outcomes encompass alteration in cell growth, changes in cell shape and morphology, variation in membrane permeability, induced gene expression, permeation of IONs into the microbial cell membrane, and the production of reactive oxygen species (ROS) [58, 60]. It has been observed that IONs do not demonstrate substantial antibacterial activity against Gram-positive bacteria, and stimulation or inhibition of growth occurs depending on the concentration of NPs [58]. Furthermore, in our previous study [54], *B. subtilis natto* (Gram-positive) growth was either stimulated or inhibited at different concentrations of uncoated IONs; however, an insignificant difference in the OD measurements between the various NP concentration groups suggested that IONs have negligible antibacterial activity against *B. subtilis natto*.

Overall, the OD values attained in the present investigation for both types of amine-functionalised IONs are substantially greater than that achieved in our earlier study [54], which explored uncoated IONs in a similar context. Ebrahiminezhad et al. [39] considered the influence of L-Lys@IONs on microbial growth, and it was determined that while bacterial immobilisation reduced cell growth by approximately 16% at the end of fermentation, the difference in the OD between the NP concentrations assessed was not significant. However, Ebrahiminezhad et al. [39] examined a lower band of L-Lys@IONs concentrations (0–150 $\upmu$g/mL) and evaluated bacterial growth using the plate count method over 5 days of fermentation. Despite these minor differences, the findings of this study are essentially similar to the current investigation, as it was determined that L-Lys@IONs do not have a significant negative impact on bacterial growth. It is also worth mentioning that the NP concentrations explored by Ebrahiminezhad et al. [39] are within the range of concentrations in this study for which a decline in the OD occurred (0–200 $\upmu$g/mL). On the other hand, Ranmadugala et al. [53] examined the effect of different concentrations of IONs@APTES (0–700 $\upmu$g/mL) on *B. subtilis* growth. It was seen that the cell density increased with an increase in the IONs@APTES concentration up to 500 $\mathrm{\upmu g}/$mL, where it reached a maximum, and a decrease in the OD was seen at higher concentrations (600 and 700 $\mathrm{\upmu g}/$mL). A similar trend in the OD measurements was observed in the present investigation, except the maximum cell density was obtained at a lower IONs@APTES concentration (200 $\upmu$g/mL). This disparity may be assigned to the different fermentation media and operating conditions employed in each study.

Moreover, it is intriguing that despite the higher cell densities attained from fermentation with the IONs@APTES in comparison to the naked IONs synthesised in our earlier investigation [54], the size of both types of NPs is the same (7–20 nm, with an average particle size of 11 nm). It is likely that the biocompatible amino acid (APTES) coating on the surface of the IONs@APTES decreases the toxicity of these NPs and results in more favourable interactions with *B. subtilis natto* cells. The L-Lys@IONs have a much smaller particle size (4–10 nm, with an average particle size of 7 nm) than the IONs@APTES and the naked IONs from our initial investigation (7–20 nm, with an average particle size of 11 nm) [54]. Certain amino acids in the reaction mixture can inhibit the growth of magnetite NPs during synthesis, resulting in smaller-sized particles, and this effect is more profound for L-Lys [57]. It has been suggested that the size of IONs influences their antimicrobial properties and toxicity towards microbial cells [59]. Smaller particles tend to have greater antimicrobial capacity, as they can easily penetrate the bacterial cell membrane relative to larger particles due to their greater surface-area-to-volume ratio, which substantially enhances their interaction with bacterial cells [59, 61, 62]. Penetration of IONs into the microbial cell can lead to membrane damage and cell inactivation through various mechanisms, primarily oxidative stress mediated by the generation of ROS (can cause DNA damage, mitochondrial impairment, lipid peroxidation, cell membrane disruption, and the oxidation of biological macromolecules), the release of metal ions (can react with the bacterial membrane and other cellular components), and the physical disruption of the cell membrane and cellular transport processes [59, 62, 63]. Nevertheless, although the L-Lys@IONs are considerably smaller than the other two types of IONs, they did not negatively affect the cell density. Also, the OD values for the L-Lys@IONs were noticeably higher than those for the uncoated IONs. This demonstrates the value of the L-Lys coating, as it reduces the antimicrobial characteristics of the NPs and significantly enhances their biocompatibility, mitigating the adverse consequences of their small particle size.

Collectively, the outcomes of the current investigation and prior studies imply that biocompatible amino acid coatings promote beneficial interactions with bacterial cells, thus reducing harmful effects on cell growth and viability. Therefore, immobilisation of *B. subtilis natto* cells with biocompatible amine-functionalised IONs does not have a negative impact on bacterial growth. Preserving the growth and metabolism of the bacteria is advantageous to enhance MK-7 synthesis and the yield of the vitamin.

### The Effect of Biocompatible IONs on the Production of All-*Trans* and *Cis* MK-7 Isomers

**Fig. 8:**
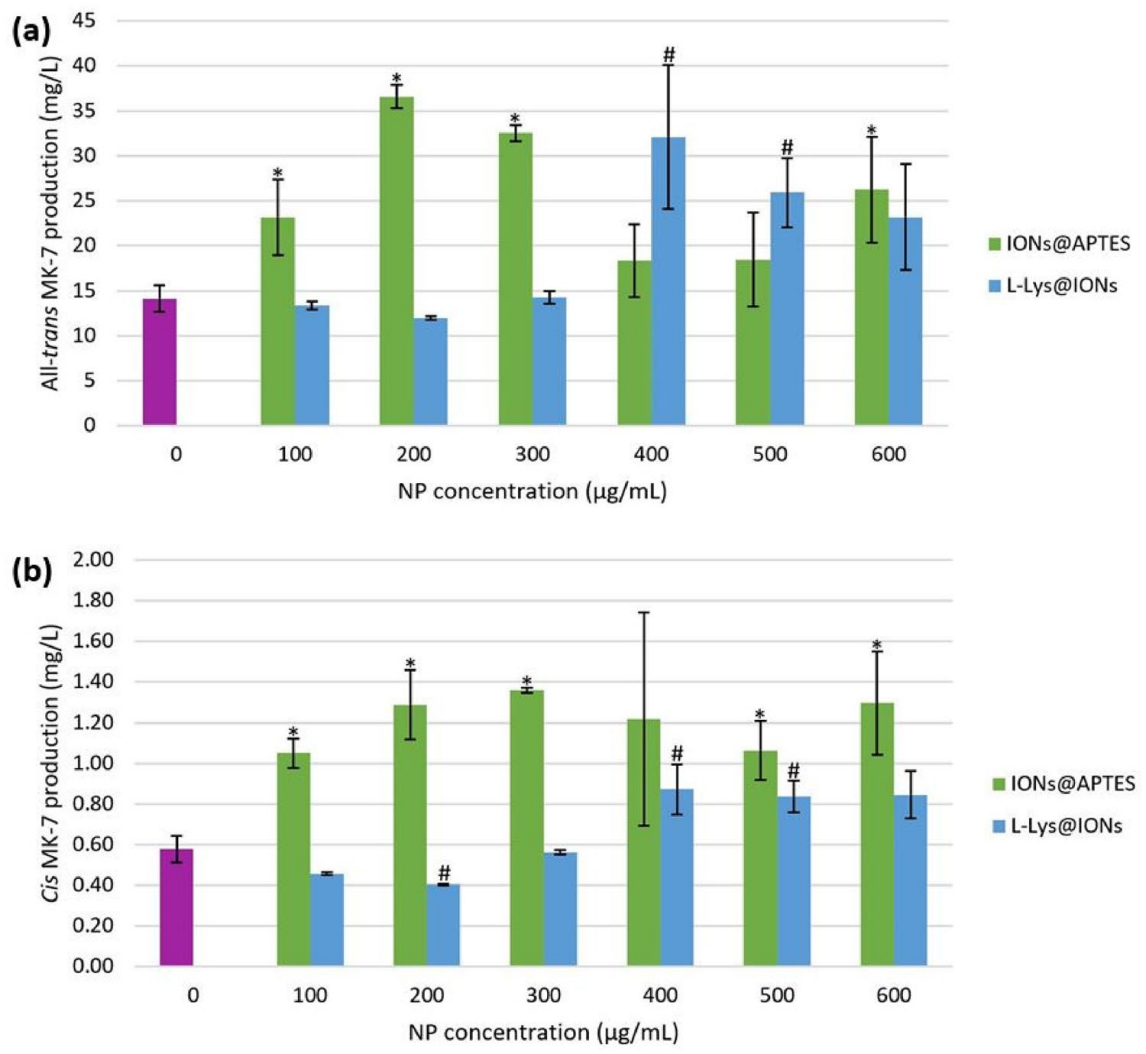
Isomer production in the presence of various concentrations of amine-functionalised IONs, where * and # indicate a significantly different **a** all-*trans* MK-7 and **b** *cis* MK-7 concentration in comparison to the control (*p* < 0.05) for the IONs@APTES and L-Lys@IONs, respectively

The influence of bacterial cell immobilisation with different concentrations of biocompatible IONs on MK-7 isomer production was assessed, and the results are outlined in Fig. 8. For the APTES-coated NPs, the concentration of the all-*trans* isomer increased with an increase in the IONs@APTES concentration to a maximum of 36.57 mg/L at a NP concentration of 200 $\mathrm{\upmu g}/$mL, and a marginally lower concentration (32.51 mg/L) was attained at an IONs@APTES concentration of 300 $\upmu$g/mL. The all-*trans* MK-7 concentration was appreciably lower for the 400 and 500 $\upmu$g/mL groups, and a slightly greater all-*trans* isomer concentration (26.24 mg/L) was obtained at the highest NP concentration. A similar trend was observed for the *cis* MK-7 concentration, except the maximum *cis* concentration was achieved at an IONs@APTES concentration of 300 $\upmu$g/mL. Conversely, for the L-Lys-functionalised IONs, a comparable all-*trans* isomer concentration was obtained at a NP concentration of 0–300 $\upmu$g/mL. Considerably greater all-*trans* MK-7 production was observed at higher concentrations of L-Lys@IONs (400–600 $\upmu$g/mL), almost twice that attained at the lower concentrations. The greatest all-*trans* MK-7 concentration (32.08 mg/L) occurred at a L-Lys@IONs concentration of 400 $\upmu$g/mL. Production of the *cis* isomer also followed a similar pattern, but the difference in the *cis* MK-7 concentration achieved between the low and high NP concentration groups was not as substantial.

The ANOVA evaluation indicated a statistically significant difference in all-*trans* MK-7 production between the different concentration groups for both kinds of amine-functionalised IONs (*p* = 0.007 for the IONs@APTES and *p* = 0.032 for the L-Lys@IONs). The ANOVA results also determined a statistically insignificant difference in the *cis* isomer concentration between the various IONs@APTES concentration groups (*p* = 0.205), while that for the L-Lys@IONs was significant (*p* = 0.003). However, it is important to appreciate that the production of both isomers in the presence of IONs@APTES was greater than the control, whereas the MK-7 isomer concentration achieved at specific concentrations of the L-Lys@IONs was less than the control. A *t*-test comparison of each NP concentration group with the control for both types of coated IONs revealed that relative to the untreated cells, all-*trans* isomer production was significantly greater for an IONs@APTES concentration of 100 $\upmu$g/mL, 200 $\upmu$g/mL, 300 $\upmu$g/mL, and 600 $\mathrm{\upmu g}/$mL and a L-Lys@IONs concentration of 400 $\upmu$g/mL and 500 $\mathrm{\upmu g}/$mL. In contrast, the *cis* MK-7 concentration was significantly higher than the control for an IONs@APTES concentration of 100 $\mathrm{\upmu g}/$mL, 200 $\mathrm{\upmu g}/$mL, 300 $\mathrm{\upmu g}/$mL, 500 $\mathrm{\upmu g}/$mL, and 600 $\mathrm{\upmu g}/$mL and a L-Lys@IONs concentration of 400 $\mathrm{\upmu g}/$mL and 500 $\mathrm{\upmu g}/$mL, while that for a L-Lys@IONs concentration of 200 $\mathrm{\upmu g}/$mL was significantly lower than the free cells.

These observations are supported by the findings of Ranmadugala et al. [53] and Ebrahiminezhad et al. [39], who have explored MK-7 production during fermentation with *B. subtilis natto* cells immobilised with IONs@APTES and L-Lys@IONs, respectively. The MK-7 concentration achieved was greater than the control for all IONs@APTES concentrations (0–700 $\mathrm{\upmu g}/$mL) considered by Ranmadugala et al. [53], whereas Ebrahiminezhad et al. [39] obtained a slightly lower MK-7 concentration for the immobilised bacteria (0–150 $\mathrm{\upmu g}/$mL) compared to the untreated cells on each day of fermentation. In addition, Ranmadugala et al. [53] noted significantly greater MK-7 production than the control for an IONs@APTES concentration of 200 $\mathrm{\upmu g}/$mL and 300 $\mathrm{\upmu g}/$mL, which is consistent with this investigation. The MK-7 concentration attained at these IONs@APTES concentrations (around 30 mg/L) is also similar to the all-*trans* isomer concentration obtained in this study for the same IONs@APTES concentrations (36.57 mg/L for 200 $\mathrm{\upmu g}/$mL and 32.51 mg/L for 300 $\mathrm{\upmu g}/$mL). Ebrahiminezhad et al. [39] achieved a final MK-7 concentration of 10.80 mg/L, 11.57 mg/L, and 11.56 mg/L for a L-Lys@IONs concentration of 50 $\mathrm{\upmu g}/$mL, 100 $\mathrm{\upmu g}/$mL, and 150 $\mathrm{\upmu g}/$mL, respectively, and all of these were marginally lower than the control (11.80 mg/L). These outcomes support the results of the current investigation for the same range of L-Lys@IONs concentrations (0–200 $\mathrm{\upmu g}/$mL). In this study, an all-*trans* MK-7 concentration of 14.13 mg/L was obtained for the free-floating cells, which was slightly higher than the cells immobilised with 100 $\mathrm{\upmu g}/$mL (13.33 mg/L) and 200 $\mathrm{\upmu g}/$mL (11.96 mg/L) of L-Lys@IONs.

Holistically, the experimental findings suggest noticeable variation in the concentration of all-*trans* MK-7 but not the *cis* isomer between all IONs@APTES concentrations examined. On the contrary, there is a considerable difference in the production of both isomers between all investigated concentrations of L-Lys@IONs. However, when evaluating the effect of bacterial cell immobilisation on the MK-7 isomer concentration by comparing the untreated cells with those immobilised with various concentrations of amine-functionalised IONs, it is apparent that certain concentrations of L-Lys@IONs impair all-*trans* MK-7 production, which is undesirable. Therefore, IONs@APTES are preferable, as production of the bioactive isomer was enhanced in comparison to the control (free cells) at all NP concentrations explored.

### The Impact of Coated IONs on the MK-7 Isomer Yield

**Fig. 9:**
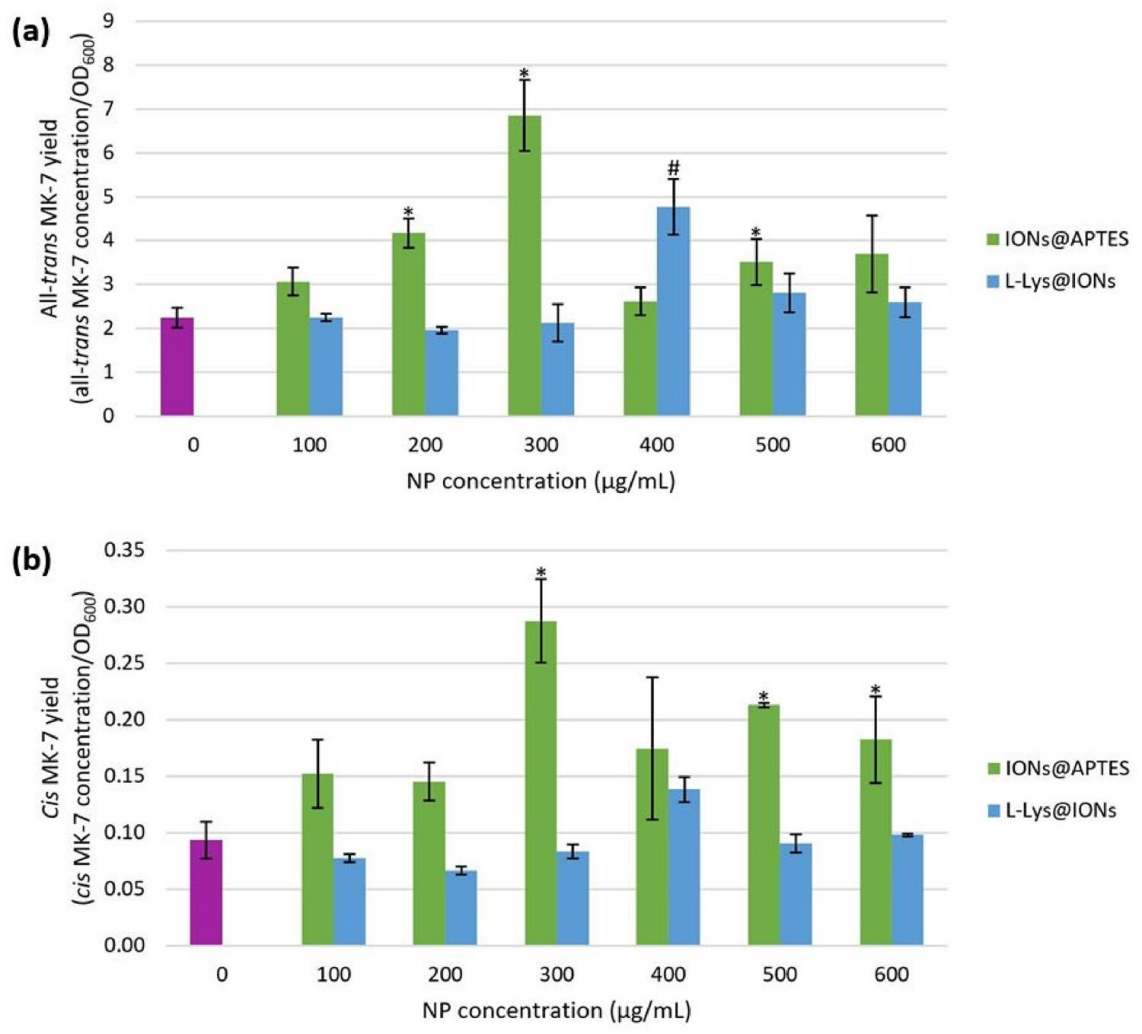
The influence of bacterial cell immobilisation with biocompatible IONs on the isomer yield, where * and # indicate a significantly different **a** all-*trans* MK-7 and **b** *cis* MK-7 yield relative to the control (*p* < 0.05) for the IONs@APTES and L-Lys@IONs, respectively

The effect of bacterial cell immobilisation with amine-functionalised IONs on fermentation productivity was evaluated by determining the isomer yield. Figure 9 illustrates the effect of the different concentrations of IONs@APTES and L-Lys@IONs on the all-*trans* and *cis* MK-7 yield. The yield of the two isomers for both types of coated IONs depicted a bell-shaped pattern. For the IONs@APTES, the isomer yield was modest for low (0–200 $\mathrm{\upmu g}/$mL) and high (400–600 $\mathrm{\upmu g}/$mL) ION concentrations, and the maximum yield was obtained at a concentration of 300 $\mathrm{\upmu g}/$mL. A similar trend was noted for the L-Lys@IONs, except the greatest yield of both isomers occurred at a concentration of 400 $\mathrm{\upmu g}/$mL.

From the ANOVA test, it was established that a statistically significant difference exists in the all-*trans* and *cis* MK-7 yield between all NP concentrations that were considered for both kinds of coated IONs (all-*trans* isomer: *p* = 0.001 for the IONs@APTES and *p* = 0.001 for the L-Lys@IONs; *cis* isomer: *p* = 0.029 for the IONs@APTES and *p* = 0.014 for the L-Lys@IONs). A *t*-test comparison of the various NP concentrations with the control for each type of amine-functionalised ION indicated that the yield of the all-*trans* MK-7 isomer was significantly higher than the untreated cells for an IONs@APTES concentration of 200 $\mathrm{\upmu g}/$mL, 300 $\mathrm{\upmu g}/$mL, and 500 $\mathrm{\upmu g}/$mL and an L-Lys@IONs concentration of 400 $\mathrm{\upmu g}/$mL. In addition, the *cis* MK-7 yield was significantly higher than the free cells for an IONs@APTES concentration of 300 $\mathrm{\upmu g}/$mL, 500 $\mathrm{\upmu g}/$mL, and 600 $\mathrm{\upmu g}/$mL, whereas, for the L-Lys@IONs, the difference in the *cis* isomer yield between the control and the remaining concentration groups was insignificant.

It is evident that generally, the yield of the bioactive isomer was higher for the IONs@APTES compared to the L-Lys@IONs. The maximum all-*trans* MK-7 yield obtained for the IONs@APTES (300 $\mathrm{\upmu g}/$mL) was 3.1-fold more than the untreated cells (0 $\mathrm{\upmu g}/$mL) and 1.4-fold greater than the highest all-*trans* isomer yield for the L-Lys@IONs (400 $\mathrm{\upmu g}/$mL). Ranmadugala et al. [53] also noted a significantly higher MK-7 yield at an IONs@APTES concentration of 200 $\mathrm{\upmu g}/$mL and 300 $\mathrm{\upmu g}/$mL; however, in contrast to our findings, the maximum yield occurred at 200 $\mathrm{\upmu g}/$mL. Ebrahiminezhad et al. [39] also observed a significantly greater MK-7 yield from fermentation with bacterial cells immobilised with L-Lys@IONs relative to the control. Furthermore, the yield of the biologically effective isomer achieved in the current investigation with 300 $\mathrm{\upmu g}/$mL of IONs@APTES and 400 $\mathrm{\upmu g}/$mL of L-Lys@IONs was 2.1- and 1.4-fold higher than that obtained for the optimum concentration of naked IONs (300 $\mathrm{\upmu g}/$mL) in our previous study [54], respectively.

It has been proposed that microbial cells immobilised with IONs have better metabolic efficiency due to the non-specific bonding associations between the NPs and the bacterial cell surface. These interactions disrupt the lipid layer of the bacterial cell membrane and increase its permeability, aiding mass transfer and enhancing the secretion of all-*trans* MK-7 into the fermentation broth [53, 59]. This improves the yield of the bioactive isomer and boosts the fermentation productivity. The superior all-*trans* isomer yield resulting from bacterial cell immobilisation with amine-functionalised IONs compared to uncoated IONs can be credited to the biocompatible APTES and L-Lys coatings, which enhance the production of biologically effective MK-7 and have a positive influence on cell growth and metabolism.

Moreover, it is worthwhile to recognise that both the production and yield of bioactive MK-7 have a direct relationship with the production and yield of the *cis* isomer. Accordingly, greater production and yield of the all-*trans* isomer correlates with higher production and yield of the *cis* isomer. While the yield of all-*trans* and *cis* MK-7 was considerably greater for the optimal concentration of IONs@APTES (300 $\mathrm{\upmu g}/$mL) and L-Lys@IONs (400 $\mathrm{\upmu g}/$mL), the fraction of the overall yield for each geometric isomer was comparable to the remaining concentration groups and the untreated cells for each type of coated ION. This indicates that although there is a noticeable difference in the isomer yield between the optimum concentration of IONs@APTES and L-Lys@IONs and the other concentration groups for both amine-functionalised IONs, the portion of the total yield for each isomer did not differ significantly. Therefore, bacterial cell immobilisation with the ideal concentration of IONs@APTES and L-Lys@IONs significantly improves the fermentation yield of the desirable isomer relative to the free cells without enhancing the proportional yield of redundant by-products, which is beneficial.

### Monitoring Study Employing the Optimal IONs@APTES Concentration

Of the two biocompatible amine-functionalised IONs that were evaluated, IONs@APTES were superior, as immobilisation of *B. subtilis natto* cells with IONs@APTES improved both the production and yield of the biologically significant isomer. Additionally, IONs@APTES have the ability to decrease biofilm formation, a prevalent issue in industrial fermentation, without any detrimental impact on cell growth and viability [64]. A comparable concentration of the all-*trans* isomer was obtained at an IONs@APTES concentration of 200 $\mathrm{\upmu g}/$mL and 300 $\mathrm{\upmu g}/$mL; however, the yield of all-*trans* MK-7 was approximately 64% greater for an IONs@APTES concentration of 300 $\mathrm{\upmu g}/$mL. Consequently, 300 $\mathrm{\upmu g}/$mL was determined to be the optimal IONs@APTES concentration to increase the production of bioactive MK-7, preserve microbial growth and metabolism, reduce biofilm formation, lessen production expenses, and enhance the efficiency of the fermentation process.

**Fig. 10:**
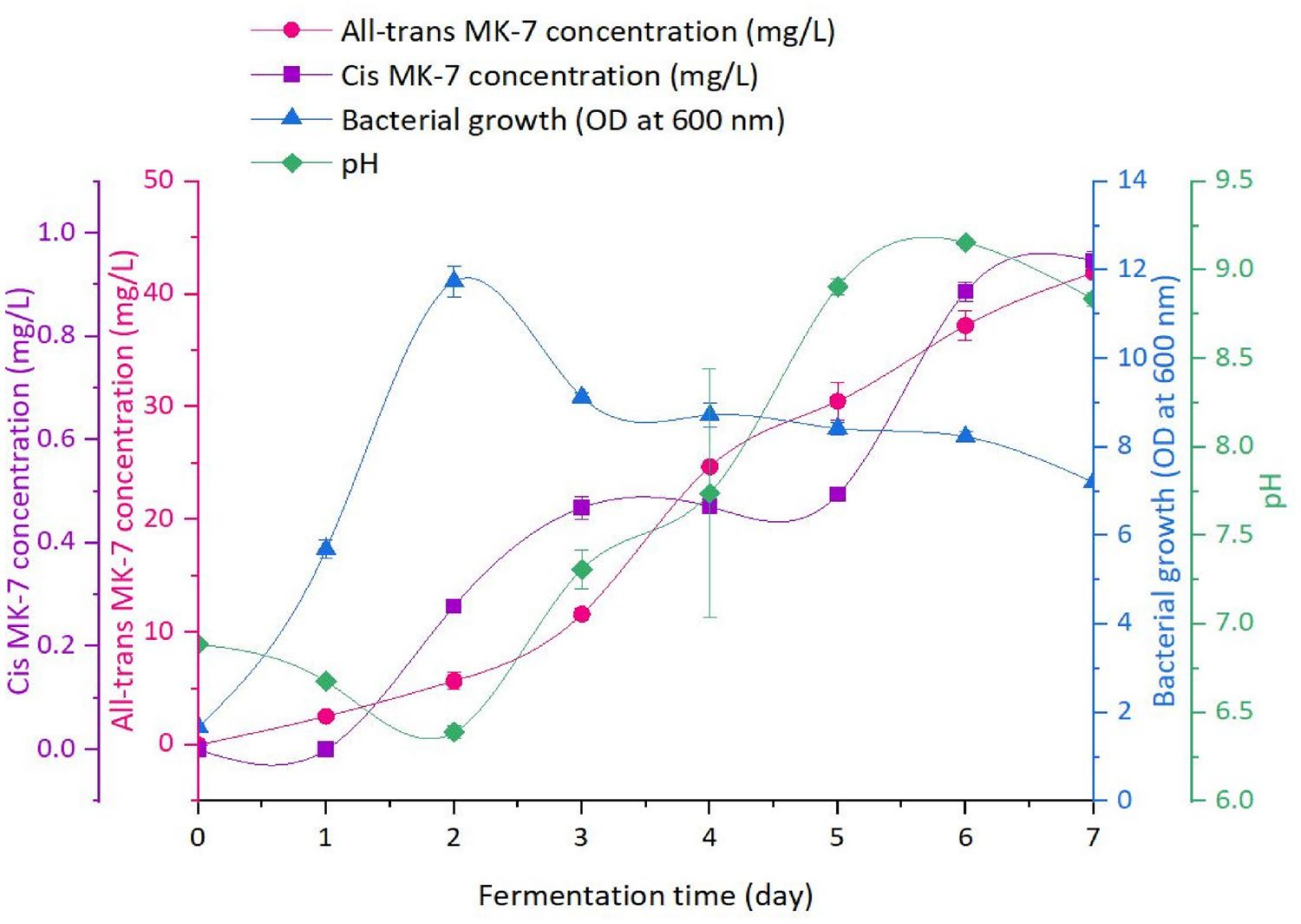
Trends in the all-*trans* and *cis* MK-7 isomer profile, microbial growth, and pH during a monitoring study utilising the optimal IONs@APTES concentration (the error bars represent the SE calculated from triplicate samples for each response)

Figure 10 demonstrates the variation in all-*trans* and *cis* MK-7 production, bacterial growth, and pH during a monitoring analysis using the optimum concentration of IONs@APTES. The trend in the OD illustrates the characteristic microbial growth profile, comprising all four stages of bacterial growth (lag, exponential, stationary, and death). The cell density progressively rose from an initial OD of 1.68 to 5.69 on day 1 before rapidly escalating to a peak value of 11.74 on day 2 of fermentation. The OD subsequently dropped to 9.13 on day 3 and levelled off (8.73–8.24) until day 6, attaining a final value of 7.21 at the conclusion of fermentation.

Production of the all-*trans* and *cis* isomer corresponded to the microbial growth curve, and the rate at which this occurred varied depending on the phase of bacterial growth. Synthesis of all-*trans* MK-7 began from day 0 and gradually increased from 0 to 11.62 mg/L until the stationary phase (day 3). The bulk of the all-*trans* isomer was produced during the stationary period (days 3 to 6), and its concentration continued to increase through to the beginning of the death phase (from day 6), reaching a value of 41.93 mg/L on day 7 of the monitoring study. In contrast, production of the *cis* isomer was first noted on day 2 (0.28 mg/L), and its concentration remained reasonably constant (0.47–0.49 mg/L) between days 3 and 5. The *cis* MK-7 concentration then rapidly increased to 0.89 mg/L on day 6, and a final concentration of 0.95 mg/L was achieved on the last day of fermentation. It appears that a minimal amount of both isomers (approximately 6.06% and 0% of the total all-*trans* and *cis* MK-7, respectively) is synthesised during the lag phase, a slightly greater concentration (around 7.50% and 29.35% of the total all-*trans* and *cis* MK-7, respectively) is observed during the exponential growth stage, and the most significant quantity (roughly 75.23% and 64.38% of the total all-*trans* and *cis* MK-7, respectively) is produced during the stationary phase. A small fraction of each isomer (about 11.21% and 6.28% of the total all-*trans* and *cis* MK-7, respectively) was also noticed during the death phase, which likely commenced on day 6. It is valuable to mention that, unlike this study, the death segment did not occur during the monitoring analyses conducted in our preceding investigations [41, 54], which employed similar fermentation conditions and also considered bacterial cell immobilisation with uncoated IONs [54]. In these studies, fermentation was usually concluded before the death stage to prevent the MK-7 in the fermentation broth from being degraded due to contact with proteases and other cellular constituents released during cell rupture. In the current investigation, it is likely that immobilisation with IONs@APTES enhanced the growth and metabolism of *B. subtilis natto,* decreasing the length of each stage of microbial growth. Thus, the death phase began earlier than our previous studies and was observed during the monitoring period. Overall, the trends and observations from this investigation are consistent with prior accounts and demonstrate that MK-7 is a mixed metabolite, as its production partially depends on bacterial growth [10, 41, 54, 65–67].

The pH of the medium varied over the time-course study, increasing from a starting value of 6.89 to 8.84 at the completion of the monitoring investigation. The rise in the medium pH during the fermentation process can be ascribed to proteolysis and the consequent release of ammonia, which occurs when the bacterium utilises proteins as a source of energy [40, 67, 68]. Despite the overall increase in the pH, it first declined to the lowest value of 6.39 on day 2 and increased to a maximum of 9.16 on day 6 before decreasing to the final value on day 7. The fluctuation in the pH can be associated with the changes in the OD and, hence, the stages of the bacterial growth curve. It is evident that the pH gradually decreased with an increase in the OD between days 0 and 2, and the lowest pH (6.39) occurred when the peak OD (11.74) was attained on day 2. The pH then increased during the stationary growth phase to the highest value (9.16) on day 6 and decreased to the final value between days 6 and 7, corresponding to the decline in the OD (death stage). The change in the pH profile and its correlation with the OD is accordant with previous reports and can be attributed to the metabolic activities of *B. subtilis natto* during the different stages of microbial growth [40, 41, 54, 67].

Ranmadugala et al. [53] also carried out a time-course fermentation study to evaluate the impact of IONs@APTES on MK-7 production, bacterial growth, and pH. While the IONs@APTES concentration differed (200 $\mathrm{\upmu g}/$mL) from the current investigation (300 $\mathrm{\upmu g}/$mL), the general trends were similar. The final MK-7 concentration achieved by Ranmadugala et al. [53] (37.36 mg/L) was slightly lower than the all-*trans* isomer concentration obtained in the present study (41.93 mg/L), indicating the excellent ability of an IONs@APTES concentration of 300 $\mathrm{\upmu g}/$mL to boost the concentration of the biologically effective MK-7 isomer. The maximum cell density reported by Ranmadugala et al. [53] (42.93) was considerably greater than that in the current investigation (11.74). This disparity may be assigned to the different IONs@APTES concentrations examined (200 $\mathrm{\upmu g}/$mL versus 300 $\mathrm{\upmu g}/$mL) and the interval over which measurements were taken during the fermentation period (every 12 h for 120 h versus every day for 7 days). Furthermore, in this study, a higher OD was achieved at the conclusion of fermentation for an IONs@APTES concentration of 200 $\mathrm{\upmu g}/$mL (8.88) than 300 $\mathrm{\upmu g}/$mL (4.96). Thus, a peak OD similar to Ranmadugala et al. [53] may have been obtained when employing an IONs@APTES concentration of 200 $\mathrm{\upmu g}/$mL for the monitoring study. However, 300 $\mathrm{\upmu g}/$mL was determined to be the optimal IONs@APTES concentration in the present investigation, as it resulted in a superior yield of the all-*trans* isomer.

In addition, the findings of this study are comparable to our earlier investigation [54], which examined uncoated IONs in a similar context, except a higher peak OD and final all-*trans* MK-7 concentration were attained from fermentation using *B. subtilis natto* cells immobilised with IONs@APTES. In particular, the maximum OD and concentration of the bioactive isomer obtained from fermentation with IONs@APTES were 19.71% and 45.66% greater than that achieved in the presence of naked IONs, respectively.

Collectively, these observations indicate that bacterial cell immobilisation with IONs containing a biocompatible APTES coating has a favourable impact on the growth and metabolism of *B. subtilis natto* and serves to enhance the metabolic efficiency of the cells, thereby increasing the concentration of the bioactive isomer and the productivity of the fermentation process.

## Conclusions

This investigation was the first to explore the effect of bacterial cell immobilisation with biocompatible amine-functionalised IONs on the concentration and yield of the all-*trans* and *cis* isomers of MK-7 achieved from fermentation. IONs@APTES and L-Lys@IONs were synthesised and characterised, and the IONs@APTES resulted in better outcomes. Immobilisation of *B. subtilis natto* cells with the optimal IONs@APTES concentration (300 $\mathrm{\upmu g}/$mL) enhanced both the production and yield of the biologically functional isomer relative to the untreated cells by 2.3- and 3.1-fold, respectively. The results of this study offer new perspectives for developing novel production methods tailored to favour the synthesis of the bioactive isomer and address the challenges of MK-7 fermentation. These advancements have the ability to streamline the fermentation system and lower manufacturing costs. This will increase the affordability of biologically efficacious MK-7 consumer end products, and their improved availability will help boost the dietary intake of all-*trans* MK-7, providing consumers with numerous health gains and easing the socioeconomic effects of an ageing global population.

## Data Availability

All relevant data that support the findings of this research are included in this article.
